# Intranasal insulin alleviates cognitive deficits and amyloid pathology in young adult APPswe/PS1dE9 mice

**DOI:** 10.1111/acel.12498

**Published:** 2016-07-26

**Authors:** Yan‐Fang Mao, Zhangyu Guo, Tingting Zheng, Yasi Jiang, Yaping Yan, Xinzhen Yin, Yanxing Chen, Baorong Zhang

**Affiliations:** ^1^ Department of Neurologythe Second Affiliated HospitalCollege of MedicineZhejiang UniversityHangzhouZhejiangChina

**Keywords:** insulin signaling, amyloid pathology, APP processing, APP/PS1, Alzheimer's disease

## Abstract

Brain insulin signaling deficits contribute to multiple pathological features of Alzheimer's disease (AD). Although intranasal insulin has shown efficacy in patients with AD, the underlying mechanisms remain largely unillustrated. Here, we demonstrate that intranasal insulin improves cognitive deficits, ameliorates defective brain insulin signaling, and strongly reduces β‐amyloid (Aβ) production and plaque formation after 6 weeks of treatment in 4.5‐month‐old APPswe/PS1dE9 (APP/PS1) mice. Furthermore, c‐Jun N‐terminal kinase activation, which plays a pivotal role in insulin resistance and AD pathologies, is significantly inhibited. The alleviation of amyloid pathology by intranasal insulin results mainly from enhanced nonamyloidogenic processing and compromised amyloidogenic processing of amyloid precursor protein (APP), and from a reduction in apolipoprotein E protein which is involved in Aβ metabolism. In addition, intranasal insulin effectively promotes hippocampal neurogenesis in APP/PS1 mice. This study, exploring the mechanisms underlying the beneficial effects of intranasal insulin on Aβ pathologies *in vivo* for the first time, highlights important preclinical evidence that intranasal insulin is potentially an effective therapeutic method for the prevention and treatment of AD.

## Introduction

Alzheimer's disease (AD) is the most common form of dementia and is characterized by progressive cognitive and functional impairment (Huang & Mucke, 2012). According to the World Alzheimer Report 2015, there are 46.8 million people living with dementia worldwide, and this is expected to increase to 74.7 million by 2030, and to as many as 131.5 million by 2050 (http://www.alz.co.uk/research/worldreport). AD has become a worldwide public health crisis. Although scientists around the world are making huge efforts, there is still no effective strategy that can cure or halt the progression of this devastating disease.

Defective insulin signaling has been observed in AD brain and is believed to be one of the etiologies for sporadic AD (Ferreira *et al*., 2014). In the central nervous system, insulin signaling regulates neuronal and glial functions such as synaptogenesis and synaptic plasticity via energy homeostasis and gene expression, and cognition (Joseph D'Ercole & Ye, 2008). Many studies have shown that patients with AD have defective insulin receptor (IR) expression and IR binding, diminished IR substrate‐1 (IRS1) and IR substrate‐2 (IRS2) expression, and increased levels of inactivated serine‐phosphorylated IRS1 (Moloney *et al*., 2010; Talbot *et al*., 2012). Furthermore, amyloid beta (Aβ) oligomers can disrupt insulin signaling through reducing surface expression of IRs in neurons or by competing with insulin (Xie *et al*., 2002). Therefore, targeting the insulin signaling pathway through delivery of insulin into the brain could be a potential therapeutic strategy to treat AD.

Intranasal drug administration is an effective method that allows therapeutic agents or neurotrophins to bypass the blood–brain barrier and directly reach the brain, thereby avoiding side effects caused by systemic delivery (Hanson & Frey, 2007). Several small‐scale clinical trials have shown that intranasal insulin improves memory and attention in healthy participants, as well as in patients with mild cognitive impairment and AD (Craft *et al*., 2012). Preclinical studies have also been conducted to confirm the cognitive improvement by intranasal insulin and the way how it reaches the brain (Marks *et al*., 2009; Salameh *et al*., 2015). However, the mechanisms underlying improved memory with insulin treatment remain unclear. We recently found that intranasal administration of insulin reduced Aβ40 levels in the 9‐month‐old transgenic AD mouse brain (Chen *et al*., 2014). To explore the role of intranasal insulin on early AD pathologies, we treated 4.5‐month‐old APPswe/PS1dE9 [amyloid precursor protein (APP)/PS1] mice, a widely used transgenic mouse model of AD, with intranasal insulin (1U) for 6 weeks. We found that intranasal insulin treatment improved cognitive deficits and insulin signaling, reduced Aβ production and amyloid plaque burden, and increased neurogenesis in young APP/PS1 mice.

## Results

### Intranasal insulin decreases anxiety‐related behaviors in APP/PS1 mice

**Figure 1:**
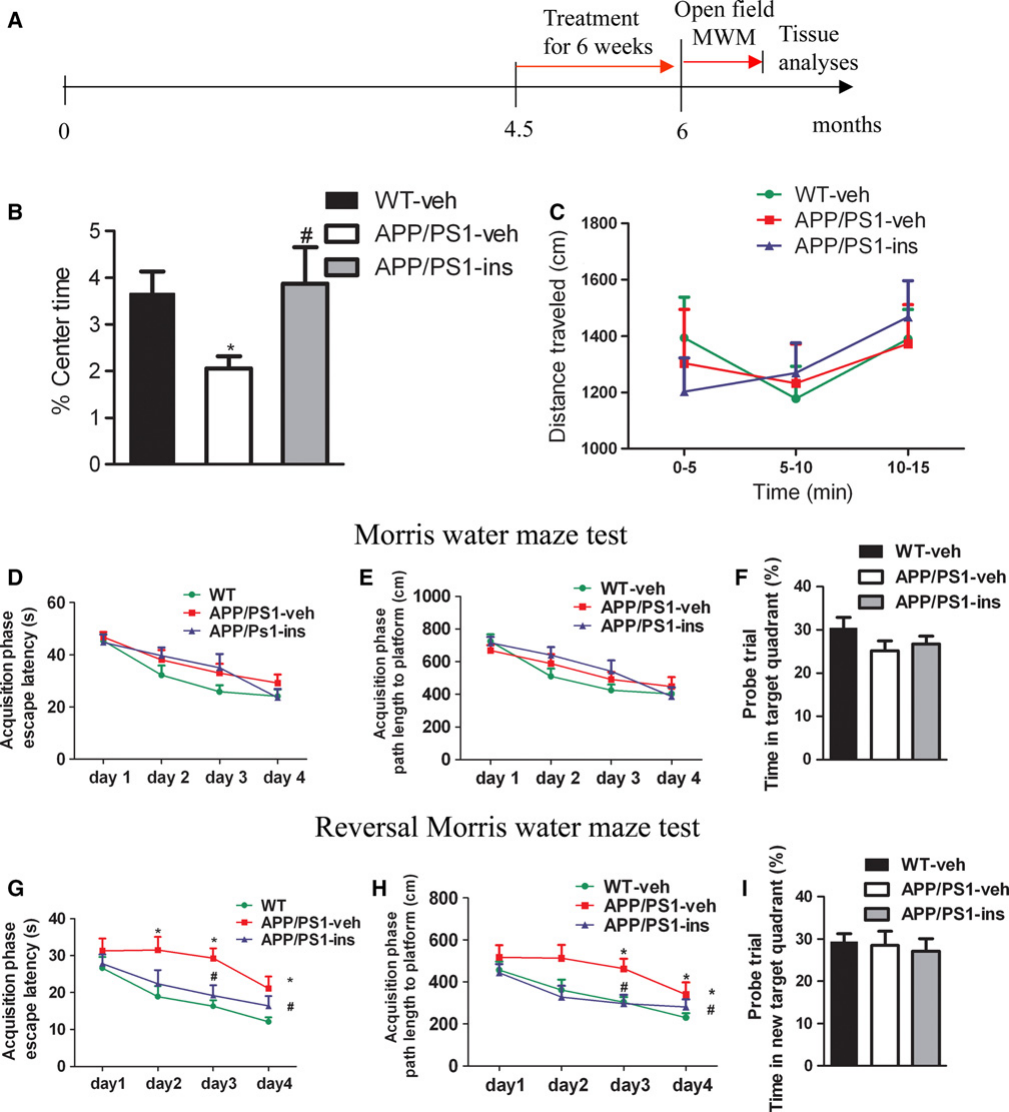
Intranasal insulin treatment attenuates anxiety‐related behaviors and improves memory plasticity in APPswe/PS1dE9 (APP/PS1) mice. (A) Animal experimental schematics. (B, C) In the open‐field test, the percentage of time spent in the center (% Center Time) (B) and the total distance traveled (C) were monitored for each animal. Insulin treatment of APP/PS1 mice increased percentage of time spent in the center, indicative of reduced anxiety. One‐way ANOVA was used for percentage of time spent in the center, and two‐way repeated‐measures ANOVA for total distance traveled, followed by Bonferroni's *post hoc* test. (D–F) Standard Morris water maze (MWM). Spatial learning was evaluated in a 4‐day acquisition phase, in which escape latencies (D) and path lengths to platform (E) were recorded. All mice exhibited a decrease in escape latencies over the 4 days of acquisition training. They had similar spatial learning function as no significant group differences in mean escape latencies or path lengths were found. Two‐way repeated‐measures ANOVA was used, followed by LSD *post hoc* test. Spatial memory was tested in probe trial in which the percentage of time spent in the target quadrant (F) was measured. No significant differences among three groups were observed, indicating indistinguishable spatial memory assessed by standard MWM. One‐way ANOVA followed by Bonferroni's *post hoc* test was performed. (G–I) To test behavioral plasticity, animals were then subjected to a reversal MWM task, in which escape latencies (G) and path lengths to platform during acquisition phase (H), and target quadrant occupancy (I) in probe trial of reversal MWM of three groups were assessed. APP/PS1 mice showed increased mean escape latencies and path lengths, while insulin treatment reversed these changes, indicating improved memory plasticity of the APP/PS1‐ins mice. The same statistics as for standard MWM were used. Values represent the mean ± SEM. *P < 0.05 vs. WT‐veh group; ^#^ *P* < 0.05 vs. APP/PS1‐veh group. WT‐veh, *n* = 17; APP/PS1‐veh, *n* = 14; APP/PS1‐ins, *n* = 13.

After 6‐week treatment with intranasal insulin, the mice were subjected to behavioral tests (Fig. 1A). During the treatment, the body weights of the mice were recorded every week. There was no significant difference in body weight among wild‐type mice treated with vehicle (0.9% NaCl) (WT‐veh), or APP/PS1 mice treated with vehicle (APP/PS1‐veh) or insulin (APP/PS1‐ins; Fig. S1). In the open‐field test, the vehicle‐treated APP/PS1 mice showed increased anxiety, as evidenced by less time spent in the center of the open field compared with the vehicle‐treated wild‐type controls. However, insulin treatment significantly alleviated anxiety in the APP/PS1 mice to a level comparable to wild‐type controls, as indicated by the increased percent of time spent in the center compared with APP/PS1‐veh mice (Fig. 1B). There was no significant difference in total distance traveled among three groups, indicating comparable locomotion in all groups (Fig. 1C).

### Intranasal insulin improves cognitive deficits in APP/PS1 mice

To investigate the spatial learning and memory of young APP/PS1 mice, the Morris water maze (MWM) test was performed. In a standard MWM test, all the mice were able to learn the platform location during the acquisition phase, as indicated by a decrease in escape latency over 4 days of acquisition training. No significant group differences in mean escape latency or path length were found (Fig. 1D,E). During the probe trial, we found no significant differences in the time spent in the former platform quadrant (Fig. 1F) or in the number of former platform site crossings among three groups (Fig. S2). These results indicated that 6‐month‐old APP/PS1 mice were indistinguishable from WT controls in spatial learning and memory assessed by MWM task. To assess the plasticity of the spatial information learned (the ability to learn a different location for the platform), which is impaired at the early phase of AD, we performed a reversal MWM (rMWM) task. During the acquisition phase, the platform was relocated, and all the mice learned to find it. However, the latency and distance traveled data indicated that vehicle‐treated APP/PS1 mice took a longer time and traveled a longer distance to reach the platform than the WT controls; intranasal insulin treatment of APP/PS1 mice led to a significant decrease in escape latency and path length traveled (Fig. 1G,H). Analysis of the reversal probe test revealed no significant difference in target quadrant occupancy among the three groups (Fig. 1I). These data indicate that 6‐month‐old APP/PS1 mice do not exhibit obvious impairments in cognitive function, as assessed by MWM test, but do show impaired spatial memory plasticity.

### Intranasal insulin improves aberrant insulin signaling in APP/PS1 mice

**Figure 2:**
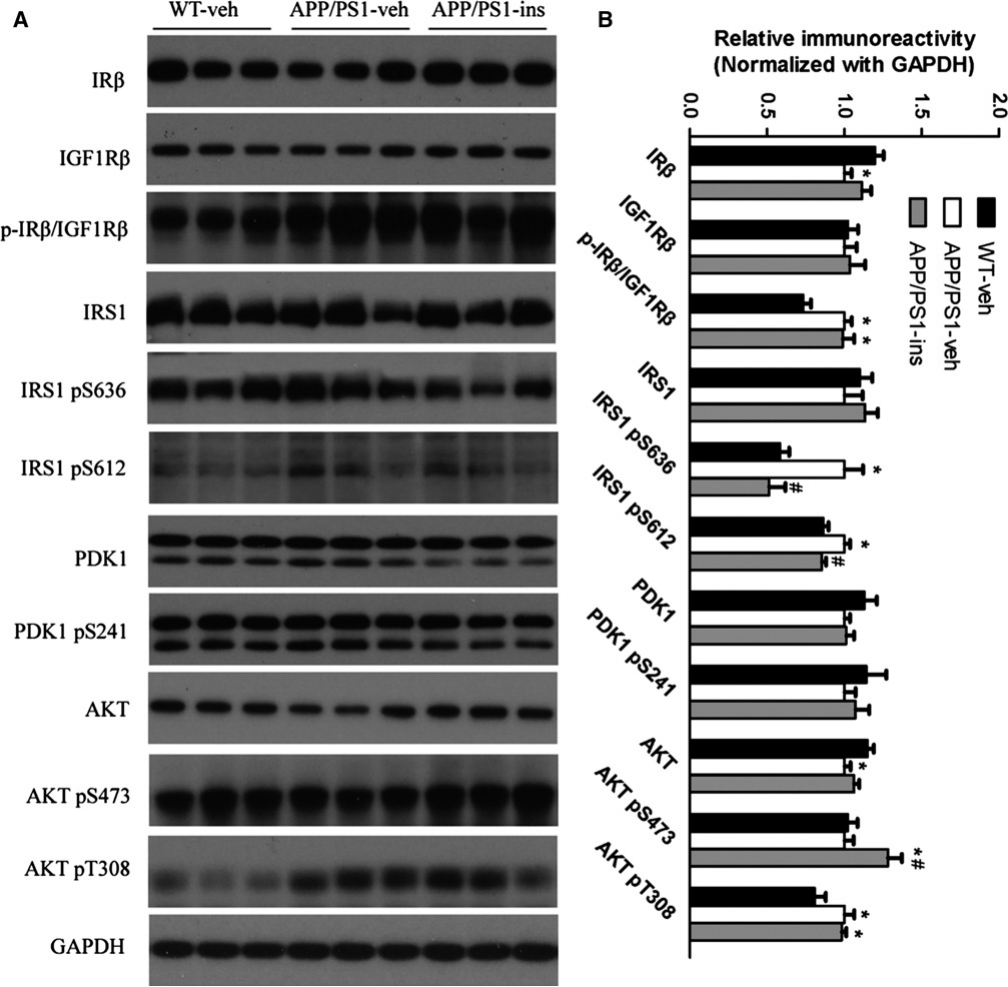
Intranasal insulin treatment improves aberrant insulin signaling in APP/PS1 mice. (A) Representative Western blots of the key insulin signaling proteins in the hippocampal homogenates from WT‐veh, APP/PS1‐veh, and APP/PS1‐ins mice. (B) Densitometric quantification of protein band optical densities for the proteins expression listed in (A). All optical densities for proteins of interest were normalized to GAPDH band densities. Data are presented as mean ± SEM. *P < 0.05 vs. WT‐veh group; ^#^ *P* < 0.05 vs. APP/PS1‐veh group, where the values of the APP/PS1‐veh group are set as 100%. *n* = 11 per group, one‐way ANOVA, followed by Bonferroni's *post hoc* test.

To investigate whether brain insulin signaling was impaired in APP/PS1 mice and whether intranasal insulin could enhance this pathway, we investigated the expression of key components involved in brain insulin signaling, including the IR β‐subunit (IRβ), type 1 insulin‐like growth factor receptor (IGF1R), IR substrate‐1 (IRS1), 3‐phosphoinositide‐dependent protein kinase‐1 (PDK1), protein kinase B (AKT), and their respective phosphorylated forms. The primary antibodies used in this study are listed in Table 1. The total levels of IRβ and AKT were significantly decreased in APP/PS1 mice, and the levels of IRS1 pS636 and IRS1 pS612, which play a central role in insulin resistance, were significantly increased in APP/PS1 mice compared with wild‐type controls. Intranasal insulin treatment promoted brain insulin signaling, as evidenced by the amelioration of the changes in the above‐mentioned proteins (Fig. 2A,B). These data indicate that there is early perturbation of brain insulin signaling in APP/PS1 mice and that intranasal insulin treatment can partially protect APP/PS1 mice from such deficits.

**Table 1:** Primary antibodies used in this study

Antibody	Phosphorylation sites	Source
IRβ		Cell Signaling Technology, Danvers, MA, USA
IGF1Rβ		Cell Signaling Technology
P‐IRβ/IGF1Rβ	Tyr1150/1151 (IRβ), Tyr1135/1136 (IGF1Rβ)	Cell Signaling Technology
IRS1		Cell Signaling Technology
IRS1 pS636	Ser636	Cell Signaling Technology
IRS1 pS612	Ser612	Cell Signaling Technology
PDK1		Cell Signaling Technology
PDK1 pS241	Ser241	Cell Signaling Technology
AKT		Cell Signaling Technology
AKT pS473	Ser473	Cell Signaling Technology
AKT pT308	Thr308	Cell Signaling Technology
GAPDH		Santa Cruz Biotechnology, Santa Cruz, CA, USA
JNK		Cell Signaling Technology
JNK pT183/pY185	Thr183/Tyr185	Cell Signaling Technology
6E10		Biolegend, San Diego, CA, USA
A11		Sigma
APP pT668	Thr668	Cell Signaling Technology
sAPPβ		IBL, Fujioka‐Shi, Gunma, Japan
sAPPα		IBL
APP C‐terminal antibody		Sigma
BACE1		Cell Signaling Technology
ADAM10		Abcam, Cambridge, MA, USA
IDE		Millipore
APOE		Santa Cruz Biotechnology
LRP1		Santa Cruz Biotechnology
Doublecortin		Santa Cruz Biotechnology

### Intranasal insulin reduces the activation of c‐Jun N‐terminal kinase in APP/PS1 mice

**Figure 3:**
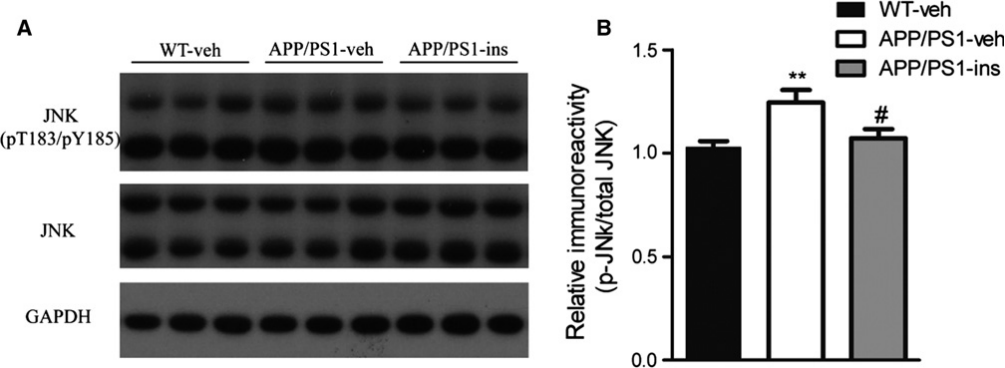
Intranasal insulin reduces the activation of c‐Jun N‐terminal kinase (JNK) in APP/PS1 mice. (A) Representative Western blots of JNK, phosphorylated JNK (p‐JNK), and GAPDH in hippocampal homogenates from the three groups. (B) Densitometric quantification of protein band optical densities for p‐JNK expressed as a ratio to total JNK level. Values are presented as mean ± SEM. **P < 0.01 vs. WT‐veh group, ^#^ *P* < 0.05 vs. APP/PS1‐veh group, *n* = 11 per group, one‐way ANOVA, followed by Bonferroni's *post hoc* test.

As c‐Jun N‐terminal kinase (JNK) plays an important role in the progression of insulin resistance and major pathologies of AD (Troy *et al*., 2001), we investigated the activation of JNK in the brain of APP/PS1 mice by assessing the level of its phosphorylation (p‐JNK) and the effects of intranasal insulin on JNK activation. We found that the level of p‐JNK was significantly increased in the hippocampus of APP/PS1‐veh mice compared with wild‐type controls, but insulin treatment decreased p‐JNK to the level of wild‐type control mice (Fig. 3). These data indicate that JNK is activated in APP/PS1 mice and that intranasal insulin can reduce its activation.

### Intranasal insulin reduces Aβ plaque deposits in the brain of APP/PS1 mice

**Figure 4:**
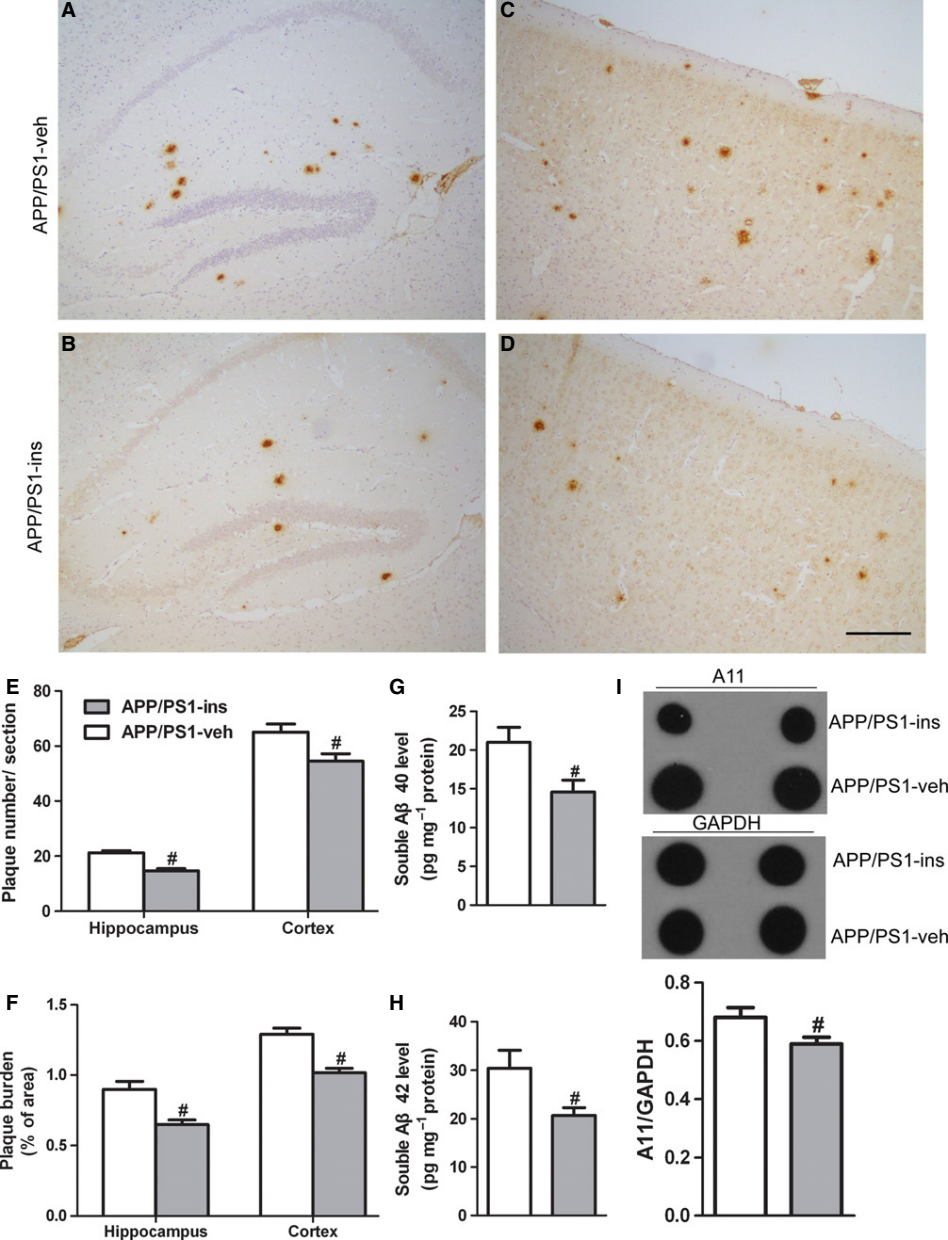
Intranasal insulin reduces Aβ plaque deposits, soluble Aβ40, Aβ42, and Aβ oligomers in the brain of APP/PS1 mice. (A–D) Representative immunohistochemical images of the hippocampus (A, B) and cerebral cortex (C, D) from APP/PS1 mice treated with vehicle (A, C) or insulin (B, D). Scale bar = 100 μm. (E, F) Statistical analyses of the number of Aβ plaques (E) and the plaque burden (F). Values are presented as mean ± SEM, ^#^ *P* < 0.05 vs. APP/PS1‐veh group, *n* = 6 per group, Student's *t*‐test. (G, H) Soluble Aβ40 (G) and Aβ42 (H) ELISAs were performed on TBS‐soluble fractions from the hippocampi of APP/PS1 mice treated with insulin or vehicle. (I) Dot blot analysis of hippocampal homogenates from the two groups using the oligomeric antibody (A11). Data are presented as mean ± SEM. ^#^ *P* < 0.05 vs. APP/PS1‐veh group, *n* = 12 per group, Student's *t*‐test.

To study the effect of intranasal insulin on Aβ pathology in APP/PS1 mice, we examined Aβ plaque deposits using immunohistochemical analysis with an antibody against Aβ (6E10). APP/PS1‐veh mice exhibited significant Aβ plaque in both the hippocampus and cortex (Fig. 4A,C). However, the number of amyloid plaques in APP/PS1 mice was reduced in both the hippocampus and cortex after insulin treatment (Fig. 4B,D,E). Changes in Aβ burden were also analyzed by measuring the areas occupied by Aβ plaques in vehicle‐ and insulin‐treated APP/PS1 mice brain. We found that the area of Aβ plaques was significantly decreased in both the hippocampus and cortex in insulin‐treated APP/PS1 mice compared with vehicle‐treated APP/PS1 controls (Fig. 4F).

### Intranasal insulin decreases Aβ levels in the brain of APP/PS1 mice

To characterize Aβ peptides present in mice brains, soluble Aβ40 and Aβ42 in hippocampal homogenates were analyzed using sandwich ELISA. In the APP/PS1‐ins mice, we detected significantly decreased soluble Aβ40 and Aβ42 levels compared with APP/PS1‐veh mice (Fig. 4G,H). Given that soluble Aβ oligomers are considered the most neurotoxic forms (De Felice *et al*., 2004), we further examined whether insulin affected the level of Aβ oligomers in the brain of APP/PS1 mice by dot blot analysis. Quantitative analysis revealed substantially reduced soluble Aβ oligomers in APP/PS1‐ins mice (Fig. 4I), confirming that intranasal insulin can decrease Aβ levels in the brain of APP/PS1 mice.

### Intranasal insulin alters APP processing in the brain of APP/PS1 mice

**Figure 5:**
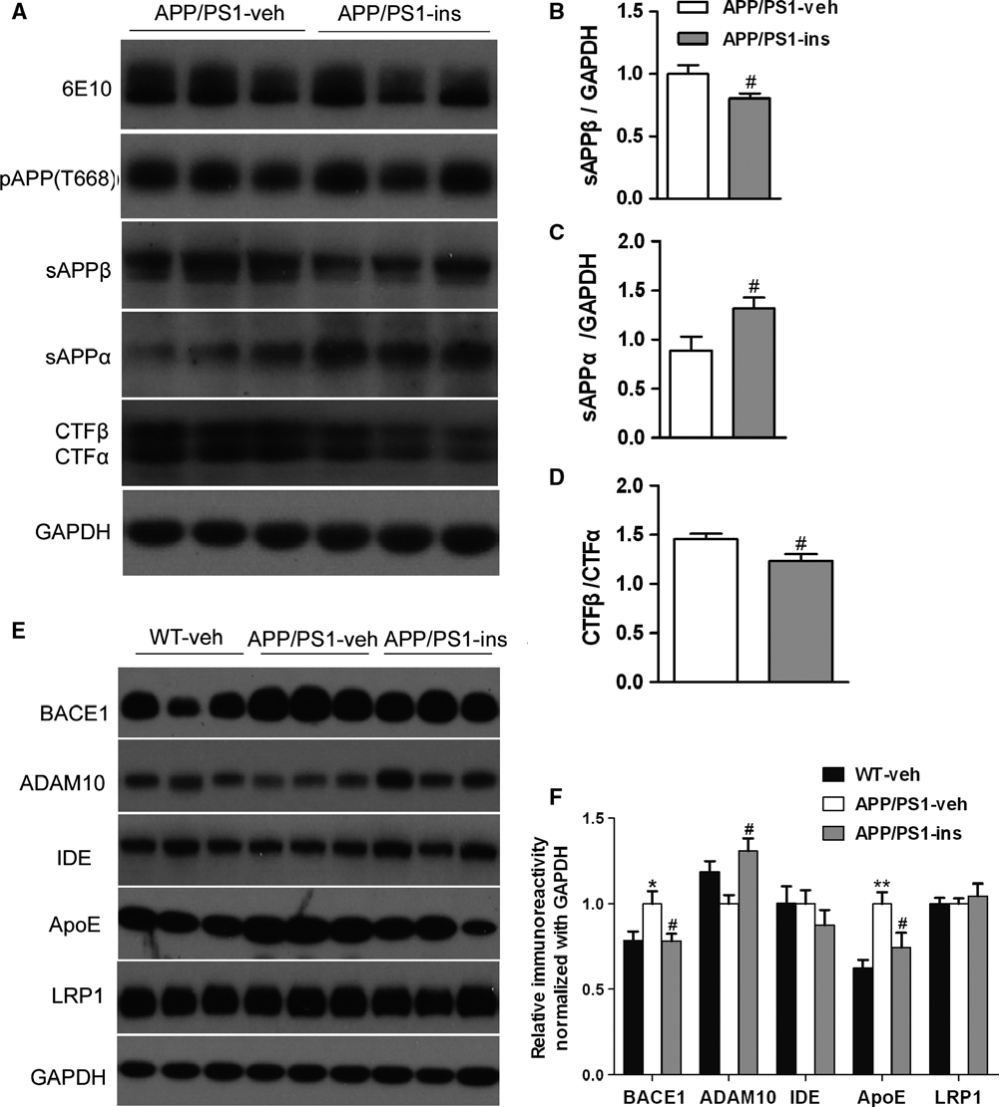
Intranasal insulin treatment alters amyloid precursor protein (APP) processing and regulates aberrant expression of BACE1, ADAM10, and Apolipoprotein E in APP/PS1 mice. (A) Representative Western blots of full‐length APP (6E10), phosphorylated APP (pT668), sAPPβ, sAPPα, and APP C‐terminal fragments (CTFα, CTFβ) in the hippocampal homogenates from APP/PS1 mice treated with insulin or vehicle. (B, C) The blots were quantified densitometrically, and the data were normalized with the GAPDH level. (D) The ratio of CTFβ to CTFα in insulin‐ or vehicle‐treated APP/PS1 mice is shown. All values are presented as mean ± SEM. ^#^ *P* < 0.05 vs. APP/PS1‐veh group. APP/PS1‐veh, *n* = 14; APP/PS1‐ins, *n* = 13, Student's *t*‐test. (E) Representative Western blots of BACE1, ADAM10, insulin‐degrading enzyme (IDE), Apolipoprotein E, LRP1, and GAPDH in the hippocampal homogenates. (F) Densitometric analysis of Western blots normalized to GAPDH, with the levels of the APP/PS1‐veh group set as 100%. Data represent mean ± SEM. *P < 0.05 vs. WT‐veh group; **P < 0.01 vs. WT‐veh group; ^#^ *P* < 0.05 vs. APP/PS1‐veh group. *n* = 11 per group, one‐way ANOVA, followed by Bonferroni's *post hoc* test.

Aβ is a product of proteolytic cleavage of APP by α‐ or β‐secretase at the extracellular region firstly, generating soluble APP fragments, sAPPα or sAPPβ, and membrane‐anchored fragments, C‐terminal fragment α (CTFα) or CTFβ, respectively. Subsequently, CTFα and CTFβ are then cleaved by γ‐secretase, producing p3 and Aβ peptides, respectively, and AICD (the APP intracellular domain). In addition to protease cleavage, phosphorylation of APP on Thr668 site facilitates the generation of Aβ (Lee *et al*., 2003). Therefore, we investigated the above APP processing products and the phosphorylation of APP on Thr668 in the hippocampus of APP/PS1 mice. Quantitative analyses of Western blots revealed that the levels of full‐length APP and p‐APP (Thr668) were comparable in hippocampal homogenates from the APP/PS1‐ins mice and APP/PS1‐veh group (Fig. 5A). However, intranasal insulin significantly decreased the production of sAPPβ and increased the level of sAPPα (Fig. 5A–C). In addition, insulin treatment resulted in a decrease in the CTFβ/CTFα ratio compared with APP/PS1‐veh mice (Fig. 5D). These data indicate that intranasal administration of insulin causes a shift toward the nonamyloidogenic pathway in the brain of APP/PS1 mice.

### Intranasal insulin regulates proteins involved in APP processing and Aβ metabolism

We then investigated the levels of APP secretases and several other proteins involved in Aβ production and metabolism. APP/PS1‐veh mice had increased cerebral levels of BACE1 protein (the major β‐secretase) compared to wild‐type controls; there was a marked reduction in BACE1 levels in APP/PS1‐ins mice (Fig. 5E,F). Conversely, there was a trend toward decreased ADAM10 (the major α‐secretase) expression in APP/PS1‐veh mice compared to wild‐type controls; insulin treatment remarkably enhanced ADAM10 level compared with the APP/PS1‐veh group. There were no changes in the level of insulin‐degrading enzyme (IDE), which is a major Aβ degradation enzyme, in the three groups (Fig. 5E,F). Apolipoprotein E (ApoE) protein has been demonstrated to play important roles in Aβ production, aggregation, and clearance (Yu *et al*., 2014). Lipoprotein receptor‐related protein 1 (LRP1), an ApoE‐binding protein, has also been identified to be involved in all these processes, contributing to the pathogenesis of AD (Harris‐White & Frautschy, 2005). We found that insulin treatment significantly restored the elevated level of ApoE found in APP/PS1‐veh mice to that of wild‐type controls. However, no significant difference in LRP1 expression was observed among the three groups (Fig. 5E,F).

### Intranasal insulin enhances neurogenesis in APP/PS1 mice

**Figure 6:**
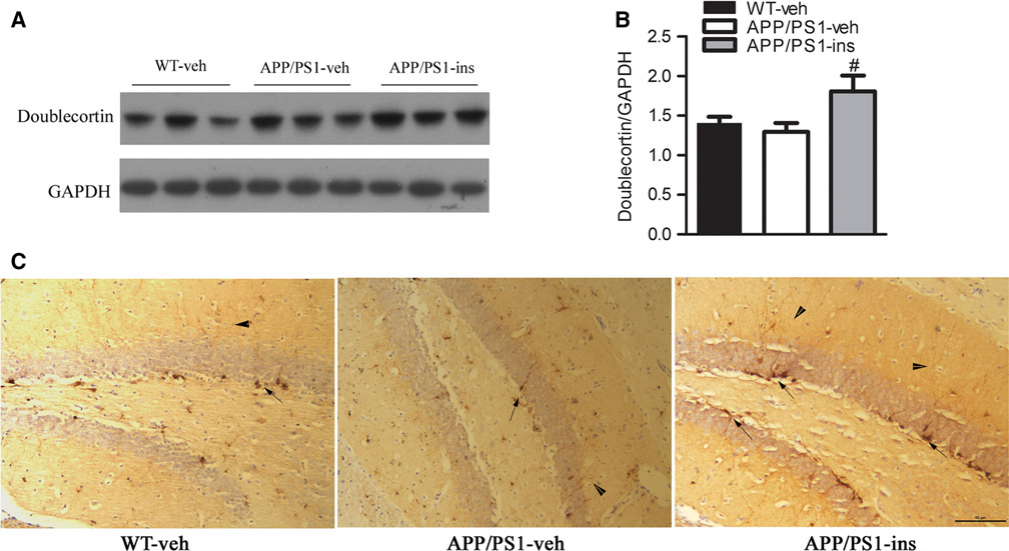
Intranasal insulin enhances neurogenesis in APP/PS1 mice. (A) Western blot analysis of doublecortin (DCX), a marker of neurogenesis, in hippocampal homogenates from WT‐veh, APP/PS1‐veh, and APP/PS1‐ins mice. (B) Densitometric quantification of the blots after normalization to GAPDH expression. Data represent mean ± SEM. ^#^ *P* < 0.05 vs. APP/PS1‐veh group, *n* = 11 per group, one‐way ANOVA, followed by Bonferroni's *post hoc* test. (C) Immunolabeling for DCX in the dentate gyrus. The number of DCX‐positive cells in the dentate gyrus of APP/PS1 mice was slightly lower than that of WT mice. The APP/PS1‐ins group had more DCX‐immunoreactive cells than APP/PS1‐veh mice. Arrow indicates soma. Arrowhead indicates extensions. Scale bar = 50 μm. *N* = 6 per group.

Hippocampal neurogenesis plays an important role in spatial learning and memory (Deng *et al*., 2010). We employed doublecortin (DCX), a marker of neurogenesis, to investigate whether intranasal insulin can also enhance the hippocampal neurogenesis. Western blot analysis revealed that the levels of DCX in the APP/PS1‐veh and WT‐veh groups were comparable. However, intranasal insulin significantly increased the level of DCX in APP/PS1 mice compared to transgenic controls (Fig. 6A,B). Immunohistochemical staining for DCX‐positive neurons in the dentate gyrus was also performed. The number of DCX‐positive cells seemed to decrease slightly in APP/PS1 mice. Intranasal insulin was able to increase the number of DCX‐immunoreactive cells in APP/PS1 mice (Fig. 6C), suggesting that insulin can enhance the neurogenesis in the hippocampus of APP/PS1 mice.

## Discussion

In the present study, we observed that 6‐month‐old APP/PS1 mice showed impairments in spatial memory plasticity. Intranasal insulin treatment improved the memory plasticity of these young APP/PS1 mice. APP/PS1 mice also exhibited increased anxiety level, as assessed by open‐field test in our study, which was alleviated by intranasal insulin treatment. These observations are consistent with a previous study showing obvious thigmotactic behavior in 7‐month‐old APP/PS1 mice of the same strain (Ferguson *et al*., 2013). However, the anxiety levels of these transgenic mice are inconsistent among different studies, and it seems that anxiety‐related behaviors are task‐specific (Lalonde *et al*., 2004; Reiserer *et al*., 2007). Other anxiety‐related tests such as light–dark test and elevated plus maze are needed to validate the anxiety level of these mice and the anxiolytic effect of intranasal insulin in these mice.

Insulin and the insulin signaling pathway have been demonstrated to be important to various functions of the brain. Physiologically, the insulin signaling pathway is activated via the binding of insulin or IGF‐I/IGF‐II to IGF1R and IR, which induce the tyrosine phosphorylation of insulin receptor substrate (IRS) and initiate the activation of the intracellular signaling pathway (White, 2006). Impaired brain insulin signaling has been well documented in patients with AD and AD animal models, and has been suggested to contribute to the cognitive deficits (Moloney *et al*., 2010) in AD. This pathological change is likely to emerge before Aβ42 accumulation (Chua *et al*., 2011). In agreement with previous findings in animal models and patients (Bomfim *et al*., 2012; Talbot *et al*., 2012), significantly reduced brain expression of IRβ and AKT and increased level of inactive IRS1 (IRS1 pS636 and IRS1 pS612) were found in the APP/PS1 mice used here. This indicates that the brain insulin signaling pathway is already compromised in 6‐month‐old APP/PS1 mice. In contrast, the levels of p‐IRβ/IGF1R and active p‐AKT (T308) were increased in these transgenic mice. We hypothesize that this may be attributed to a compensatory response against early insulin signaling defects (Moloney *et al*., 2010). With intranasal insulin treatment, the levels of IRβ and AKT in these APP/PS1 mice showed a trend toward returning to the level of wild‐type controls. Serine phosphorylation in IRS1 (IRS1pSer) has been found in the brain of the APP/PS1 transgenic mouse model and in hippocampi of cynomolgus monkeys receiving i.c.v. injections of Aβ oligomers (Bomfim *et al*., 2012). Inhibitory IRS1pSer can block downstream insulin signaling (Zhao *et al*., 2008b). Furthermore, the levels of p‐IRS1 (pS636) and p‐IRS1 (pS612) are positively correlated with Aβ plaques and negatively associated with episodic and working memory (Talbot *et al*., 2012). In this study, we found significantly increased p‐IRS1 (pS636) and p‐IRS1 (pS612) in vehicle‐treated transgenic mice; these are considered a key signature of insulin resistance (Talbot *et al*., 2012). These increases were downregulated by insulin administration. It is plausible that intranasal insulin treatment can ameliorate insulin resistance due to increased IRS1pSer, leading to a reduction in Aβ production and amyloid plaque burden, and improvement of cognition.

c‐Jun N‐terminal kinase is one of the major kinases that can inhibit insulin signaling by modulating the phosphorylation of IRS proteins (Zick, 2005). P‐JNK staining is found to localize in neurons surrounding amyloid plaques and in neurons that contained intracellular accumulations of Aβ both in patients with AD and in AD mouse models (Shoji *et al*., 2000; Zhu *et al*., 2001). The levels of JNK3 and p‐JNK are also increased in cerebrospinal fluid (CSF) and are correlated with the rate of cognitive decline in patients with AD (Gourmaud *et al*., 2015). In primary hippocampal neurons, the levels of active JNK and phosphorylated IRS1 (Ser616) and tau (Ser422) are significantly increased by exposure to Aβ oligomers (Shoji *et al*., 2000). Conversely, JNK inhibition is capable of reducing Aβ toxicity and ameliorating the major pathological features of AD (Troy *et al*., 2001; Zhou *et al*., 2015). In the present study, we found that intranasal insulin was revealed to target JNK activation in APP/PS1 mice. This may contribute to the beneficial effects of intranasal insulin in rescuing defective insulin signaling and Aβ‐related pathologies.

Given that accumulation of Aβ in the brain is the major pathological feature of AD, we investigated whether intranasal insulin could affect Aβ deposits and soluble Aβ level in APP/PS1 mice. We found a reduction of Aβ plaques and soluble Aβ (Aβ40 and Aβ42) after insulin treatment in APP/PS1 mice. This was consistent with our recent preliminary study showing that insulin decreased Aβ40 levels in the brains of 3xTg‐AD mice (Chen *et al*., 2014). Notably, soluble Aβ oligomers, which are powerful neurotoxins, were also found to decrease after insulin treatment.

We further investigated factors related to Aβ production and metabolism. BACE1 is the major β‐secretase for Aβ generation in neurons (Cai *et al*., 2001). Targeting BACE1 can rescue memory deficiency and ameliorate AD neuropathologies (Singer *et al*., 2005). ADAM10 is the major α‐secretase that cleaves APP to sAPPα (Kuhn *et al*., 2010). In line with a previous study (Gallagher *et al*., 2013), we found that expression of BACE1 was increased in APP/PS1‐veh mice compared with wild‐type controls. Intranasal insulin caused a shift to the nonamyloidogenic, α‐secretase‐dependent APP processing pathway, as demonstrated by increased ADAM10 expression and decreased BACE1 levels in APP/PS1‐ins mice compared with the APP/PS1‐veh group.

The *APOE* gene on chromosome 19 has three common alleles (*APOE* ε2, *APOE* ε3, and *APOE* ε4), which encode three major isoforms. People who carry *APOE* ε4 allele have an increased risk of developing AD, while *APOE* ε2 carriers are protected from the disease (Deelen *et al*., 2011). The level of soluble ApoE protein increases both in patients with AD and in APP/PS1 rat model of AD (Arold *et al*., 2012). It plays an important role in Aβ aggregation and fibril formation (Wisniewski *et al*., 1994). In addition, human ApoE haploinsufficiency reduces amyloid deposition in an AD mouse model (Kim *et al*., 2011). In the present study, intranasal insulin decreased the elevated level of ApoE found in APP/PS1‐veh mice. This may be another factor that contributed to reduction in Aβ plaque and oligomers of APP/PS1‐ins mice. The levels of two other proteins related to Aβ metabolism, IDE and LRP1, were unchanged. We did not find any change in APP processing‐related APP phosphorylation. These results suggest that the amelioration of cerebral amyloidosis in intranasal insulin‐treated APP/PS1 mice probably results from enhanced nonamyloidogenic APP processing, decreased amyloidogenic BACE1 cleavage, and reduced expression of ApoE protein.

Extensive evidence has shown that new neurons generated in the dentate gyrus of the adult mammal are important in learning and memory (Deng *et al*., 2010). Physical exercise and enriched environment promote hippocampal neurogenesis, whereas aging and stress are two major inhibitors of neurogenesis (Zhao *et al*., 2008a). A previous study showed that neurogenesis is slightly but insignificantly impaired in APP/PS1 mice at the age of 5 months compared to controls (Hamilton & Holscher, 2012). Our findings in 6‐month‐old APP/PS1 mice are consistent with this study. Intranasal insulin significantly promoted neurogenesis in the brain of APP/PS1 mice. IGF1 is a widely researched hormone that mediates neurogenesis (O'Kusky *et al*., 2000), and it is possible that intranasal insulin may enhance neurogenesis via the same mechanism as IGF1, which deserves further confirmation.

In conclusion, intranasal insulin treatment for 6 weeks can effectively decrease anxiety‐related behaviors and ameliorate cognitive impairments, impaired brain insulin signaling, and neurogenesis, and substantially decrease brain Aβ level and Aβ plaque deposits by shifting APP processing to the nonamyloidogenic pathway and causing a reduction in ApoE protein in young adult APP/PS1 mice. These findings suggest that the beneficial effects of intranasal insulin on cognition observed both in animal models and in clinical trials could at least partially be attributable to the enhancement of insulin signaling, alleviation of Aβ pathology, and promotion of neurogenesis. Our study further provides the mechanistic basis for the treatment of AD with intranasal insulin.

## Experimental procedures

### Antibodies and reagents

The primary antibodies used in this research are listed in Table 1. Peroxidase‐conjugated secondary antibodies were obtained from Santa Cruz Biotechnology (Santa Cruz, CA, USA). Insulin (Humulin R) was purchased from Eli Lilly (Indianapolis, IN, USA). All other chemicals were obtained from Sigma (St. Louis, MO, USA).

### Animals and treatments

Homozygous APPswe/PS1dE9 (APP/PS1) double‐transgenic mice harboring human APPswe (Swedish mutations K594N/M595L) and presenilin‐1 with the exon 9 deletion (PS1dE9) under the control of the mouse prion protein promoter were used in this study and were obtained from The Jackson Laboratory [strain name, B6C3‐Tg (APPswe, PSEN1dE9)85Dbo/J; stock number 004462]. All female APP/PS1 transgenic mice (4.5 months old) with a confirmed genotype were produced by the Model Animal Research Center of Nanjing University (Pan *et al*., 2010). Female transgenic mice were used because female mice manifest an early deficit in spatial memory in the MWM test and develop greater Aβ burden compared to age‐matched male mice (Gallagher *et al*., 2013). The 4.5‐month‐old mice were selected because this transgenic line develops amyloid plaque by 4–6 months of age (Yan *et al*., 2009), and memory deficits as early as 6 months of age by contextual fear conditioning test (Kilgore *et al*., 2010). The 4.5‐month‐old mice are suitable for investigating the effect of intranasal insulin on early Aβ pathology. The use and care of the mice complied with the guidelines of the Animal Advisory Committee at Zhejiang University and the US National Institutes of Health Guidelines for the Care and Use of Laboratory Animals.

APP/PS1 mice and their nontransgenic wild‐type littermates were randomized into three groups: vehicle (0.9% NaCl)‐treated wild‐type mice (WT‐veh), *n* = 19; vehicle‐treated APP/PS1 mice (APP/PS1‐veh), *n* = 15; and insulin‐treated APP/PS1 mice (APP/PS1‐ins), *n* = 15. As the objective of our study was to investigate the underlying mechanism by which intranasal insulin benefits the AD mouse model, we did not include a group of wild‐type mice treated with insulin. Mice were housed four animals per cage, with free access to food and water, and maintained on a 12/12‐h light/dark cycle in a temperature‐controlled room (22 °C). Intranasal delivery of insulin or vehicle was carried out manually without anesthesia while the mouse's head was restrained in a supine position with the neck in extension, as previously described (Hanson *et al*., 2013). Briefly, the mice were habituated to handling for 14 days prior to start of the experiment. A total of 1 U/25 μL insulin or vehicle per day was delivered over both nares, 3.0 μL each time into alternate nostrils. After 6 weeks of treatment with intranasal insulin, the mice were subjected to behavioral tests including open‐field and MWM tests. Finally, the mice were killed by decapitation, and the brains were removed immediately and cut sagittally into left and right hemispheres. The right hemisphere was fixed in 4% paraformaldehyde, and routine paraffin sections (7 μm) were prepared for immunohistochemistry analyses. The hippocampus and cerebral cortex were dissected from the left hemisphere, flash‐frozen separately in dry ice, and stored at −80 °C for biochemical analyses.

### Open‐field and Morris water maze tests

Anxiety and exploratory activities were analyzed using an open‐field test. The test arena was constructed of a plastic chamber (81 × 81 cm, with 28.5‐cm‐high walls). A video camera connected to a computer was placed above the chamber to monitor the distance traveled and the time spent in the center and peripheral area in the arena. Every mouse was placed in the center of the arena and allowed to explore for 15 min. Before each trial, the surface of the box was cleaned and dried.

One day after the open‐field test, the spatial learning and memory of the mice were evaluated in the MWM test. Briefly, the test was performed in a white pool 100 cm in diameter filled with water tinted with nontoxic white paint and maintained at room temperature (21 ± 2 °C). During the acquisition phase, an escape platform (10 cm in diameter) was placed 1 cm below the water surface in one quadrant of the pool. The environment surrounding the pool was decorated with geometric objects as spatial cues. Each mouse was subjected to four trials a day for four consecutive days. Each trial began by placing the mouse randomly into a position in one of the four quadrants of the pool and allowing it to swim freely for a maximum of 60 s. After locating the platform (or being guided to the platform if the mouse failed to reach the platform after 60 s), the animal was allowed to stay on the platform for 15 s. The time for each animal to locate the platform in all four trials (escape latency) and the path length swimming to platform were recorded. Twenty‐four hours after the acquisition phase, a 60‐s probe trial was performed to determine memory retention. During the probe trial, each mouse was started in the position opposite to the quadrant that formerly contained the platform in acquisition testing. The animal's swim path, the percent of time spent in the target quadrant, average swim speed, and platform site crossings were recorded with a video tracking system (Actimetrics, Wilmette, IL, USA).

After 5 days of standard MWM, a rMWM task was performed. This involved moving the location of the escape platform diagonally, followed by a 4‐day acquisition phase, with a probe trial on day 5. The same parameters as for the standard MWM were recorded with the video tracking system.

### Immunohistochemistry and histology

Immunohistochemical staining was performed to examine the distribution of Aβ plaques and neurogenesis in the APP/PS1 mice brain. Briefly, paraffin sections were deparaffinized, rehydrated, and then boiled in citric acid buffer for 10 min. After washing with TBS buffer, sections were incubated in 3% H_2_O_2_ to quench endogenous peroxidase activity. Sections were then rinsed, incubated in 5% normal goat serum in TBS (50 mm Tris‐HCl, pH 7.4, 150 mm NaCl) for 30 min, and incubated overnight at 4 °C with mouse anti‐Aβ antibody 6E10 (1:500) or anti‐DCX (1:60). Primary antibody was detected with horseradish peroxidase‐conjugated secondary antibody and visualized with a stable diaminobenzidine solution. The stained sections were dehydrated through graded alcohols, cleared in xylene, and covered with neutral balsam. For quantification analyses, five sections with the same reference position were selected from each mouse (*n* = 6) to assess the average plaque number and Aβ burden using image‐pro plus imaging software (Media Cybernetics, Bethesda, MD, USA).

### Western blot analyses

Hippocampi were homogenized in chilled TBS supplemented with protease and phosphatase inhibitor cocktail (Thermo Fisher Scientific, MA, USA). Protein concentrations of the homogenates were determined by bicinchoninic acid (BCA) protein assay (Thermo Fisher Scientific). Samples were electrophoretically separated on 10% or 12% SDS‐PAGE gels and transferred to PVDF membranes (Millipore, Bedford, MA, USA). Membranes were blocked with 5% nonfat milk in TBS containing Tween‐20 (TBS‐T) (0.1% Tween‐20 in TBS buffer) for 1 h and probed with primary antibodies at 4 °C overnight. The membranes were then developed with the corresponding horseradish peroxidase‐conjugated secondary antibody and ECL kit (Thermo Fisher Scientific). Densitometric quantification of protein bands in Western blots was analyzed using the multi gauge V3.0 software (Fuji Photo Film Co., Ltd, Edison, NJ, USA).

### Dot blot assays

In this assay, soluble proteins were extracted from brain samples (hippocampus) with TBS in the presence of protease and phosphatase inhibitors and centrifuged at 13 000 *g* at 4 °C for 30 min. A total of 3 μL of the sample was directly applied to nitrocellulose membrane (Millipore), air‐dried, and blocked with 5% nonfat milk. The membrane was then incubated with oligomer‐specific A11 antibody at 4 °C overnight and processed as described above for Western blots.

### ELISA for total soluble Aβ in brain extracts

Soluble Aβ40 and Aβ42 levels were measured using ELISA kits (Invitrogen Life Technologies, Carlsbad, CA), according to the manufacturer's instructions. Briefly, frozen hippocampi of vehicle and insulin‐treated APP/PS1 mice were homogenized in ice‐cold TBS supplemented with protease and phosphatase inhibitor cocktail (Thermo Fisher Scientific). Brain homogenates were centrifuged at 13 000 *g* at 4 °C for 30 min. The supernatant was then diluted 1:10 before the ELISA was carried out. Protein was quantified using the BCA protein assay. All ELISA assays were carried out with the titrated sample amounts that yielded OD values falling within the quantifiable range of the assay. The Aβ values were then converted to pg mg^−1^ protein.

### Statistics

Differences between means were analyzed using either two‐way repeated‐measures ANOVA or one‐way ANOVA followed by Bonferroni's *post hoc* analysis, or Student's *t*‐test when indicated. All data are expressed as means ± SEM, and *P* < 0.05 was considered statistically significant. All statistical analyses were performed using Prism (GraphPad Software Inc., San Diego, CA, USA).

## Funding

This work was supported by grants from the National 973 Project (grant numbers 2013CB530900, 2013CB530904), the National Nature Science Foundation of China [grant numbers 81400866, 81200984], the Projects of International Cooperation and Exchanges NSFC [grant number 81520108010], and the New Doctorate Teacher Fund from Ministry of Education of China [grant number 20120101120036].

## Conflict of interest

None declared.

## Author contributions

Y.M., Z.G., Y.C., and B.Z. designed the study, performed most of experiments, and wrote the manuscript. T.Z. and Y.J. performed other experiments. Y.Y. and X.Y. provided critical reagents. All authors reviewed the results and approved the final version of the manuscript.

## Supporting information


**Fig. S1** The body weights of the three groups were not significantly different during the treatment.
**Fig. S2** In probe trial of stand Morris water maze test, the numbers of former platform site crossings are not significantly different among three groups.
