# Supplementary material for: Intranasal insulin alleviates cognitive deficits and amyloid pathology in young adult APPswe/PS1dE9 mice
Source: Aging Cell. 2016 Jul 26;15(5):893–902. doi: 10.1111/acel.12498 (PMC5013027; doi:10.1111/acel.12498)
Supplement: Supplementary file 1 — Fig. S1 The body weights of the three groups were not significantly different during the treatment. Fig. S2 In probe trial of stand Morris water maze test, the numbers of former platform site crossings are not significantly different among three groups. [file ACEL-15-893-s001.docx]

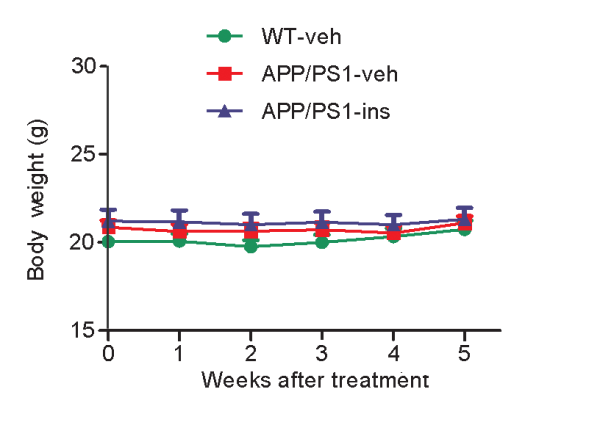


Fig. S1 The body weights of the three groups were not significantly different during the treatment.


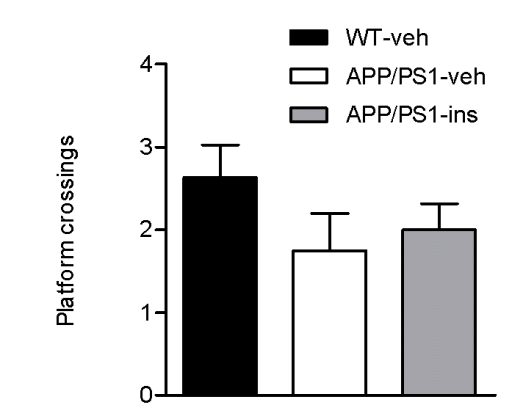


Fig. S2 In probe trial of stand Morris water maze test, the numbers of former platform site crossings are not significantly different among three groups.
